# Nano-Sized Antioxidative Trimetallic Complex Based on Maillard Reaction Improves the Mineral Nutrients of Apple (*Malus domestica* Borkh.)

**DOI:** 10.3389/fnut.2022.848857

**Published:** 2022-04-25

**Authors:** Yu-zhang Yang, Qin-ping Wei, Jia Zhou, Min-ji Li, Qiang Zhang, Xing-liang Li, Bei-bei Zhou, Jun-ke Zhang

**Affiliations:** Institute of Forestry and Pomology, Beijing Academy of Agriculture and Forestry Sciences, Beijing, China

**Keywords:** trimetallic complex, Maillard reaction, antioxidative activity, fruit quality, mineral nutrients

## Abstract

The metallic complex is widely used in agricultural applications. Due to the oxidation of the metal and environmental unfriendliness of ligand, maintaining an efficient mineral supply for plants without causing environmental damage is difficult. Herein, an antioxidative trimetallic complex with high stability was synthesized by interacting Ca^2+^, Fe^2+^, and Zn^2+^ with the biocompatible ligands from the Maillard reaction. The composite structure elucidation was carried out by transmission electron microscopy (TEM), scanning electron microscopy (SEM), X-ray photoelectron spectroscopy (XPS), and Fourier transform infrared (FTIR). Thermal stability was measured by thermogravimetric (TG). Antioxidative activities were evaluated by ferric reducing antioxidant power and radical scavenging activity assays. The three metals were successfully fabricated on the Maillard reaction products (MRPs) with contents of Ca (9.01%), Fe (8.25%), and Zn (9.67%). Microscopy images revealed that the three metals were uniformly distributed on the MRPs with partial aggregation of <30 nm. FTIR and XPS results revealed that the metals were interacted with MRPs by metal–O and metal–N bonds. TG and antioxidative activity assays showed that the trimetallic complex meets the requirements of thermodynamics and oxidation resistance of horticultural applications. Additionally, the results of the exogenous spraying experiment showed that the trimetallic complex significantly increased the mineral contents of the “Fuji” apple. By treatment with the complex, the concentrations of Ca, Fe, and Zn were increased by 85.4, 532.5, and 931.1% in the leaf; 16.0, 225.2, and 468.6% in the peel; and 117.6, 217.9, and 19.5% in the flesh, respectively. The MRP-based complexes offered a higher growth rate of the mineral content in apples than ones based on sugars or amino acids. The results of the spraying experiment carried out in 2 years show that the method has high reproducibility. This study thus promotes the development of green metallic complexes and expands the scope of agrochemical strategy.

## Introduction

Mineral nutrients, generally deemed metal elements, benefit the storage, disease prevention, bioactivity, and internal quality of fruits (1–4). However, metal ions form insoluble metal oxides and consequently tend to persist in soil rather than be absorbed by plants (5, 6), and the characteristics of facile oxidation and precipitation of metal elements are amplified in the alkaline and calcareous environment which covers more than 30% of the global land surface (7, 8). Supplements and improvement in the content of mineral nutrients of fruits are subject to soil conditions; therefore, it requires human management in the orchards. Existing strategies take advantage of extraneous spraying of mineral nutrients. However, the application of soluble minerals is inefficient because a significant portion of metal ions is oxidized before they go into the plants. Conventional approaches for augmenting the stability of mineral elements rely on the use of metallic complex which is structured with synthetic or biological ligands (9–11). The ligands offer oxygen- or nitrogen-containing groups to form coordination bonds with metal ions to improve ion stability. Although metallic complexes can address these problems to a certain extent, many metallic complexes are either inefficient or costly and may adversely affect the environment (12–14). Therefore, developing biodegradable, eco-friendly, and absorption-promoted ligands of mineral elements are desirable for biological applications.

Antioxidative materials have attracted tremendous research interest in the field of food and medical engineering owing to their ability to construct carrier structures that can protect and deliver payloads into specific receptors (15–17). Of these, Maillard reaction products (MRPs) produced through the Maillard reaction (MR), which is a non-enzymatic browning reaction and widespread in food processing involving carbonyl and amino compounds (18–20), are efficient ligands for capturing metal ions for application in food additives and drug delivery (21, 22). The complicated structures of MRPs supply numerous binding sites with metal ions under mild conditions, thus offering a potential method for simultaneously capturing multiple metals, such as Ca^2+^, Fe^2+^, Zn^2+^, Cu^2+^, and Mg^2+^, to improve their solubility (23). Previously, we have proved that the alkaline stability of Fe^2+^ can be improved by MRPs (24). The polymerized fraction of MRPs generated under alkaline conditions has the capability to turn the interaction of MRPs and Fe^2+^ from low binding affinity to high binding affinity by constructing more Fe–N bonds. Moreover, MRPs have been proven to be antimicrobial and biodegradable for biological applications (25–29). Thus, we suggested that MRPs have great potential in agricultural applications because they are not only an antioxidative ligand that can capture metals and prevent their oxidation but also a biocompatible carrier that guarantees sustained mineral supply for plants.

In contrast, the efficient transport of minerals to plants requires further resolution. Minerals move passively through the transpiration flux from the roots. However, fruits have a low transpiration surface in relation to their volume, and their mobility of nutrients through the phloem, which transports sugars from leaves to developing fruits, is ineffective (30). The limited mobility makes it necessary that spraying minerals on plants are applied directly since apparent mineral nutrients are not easily re-translocated from other organs to the fruit (31–34). Many studies have been carried out to study the spray effect of metal salts (35–37), metal oxides (38, 39), and metal complexes (40–43) on plant growth. Ca sprays are more effective than soil applications at allocating and increasing the Ca content in the apple fruits and could help reduce the incidence of Ca-related disorders (44, 45). Zn sprays enhance the fruit Zn content and result in the higher activities of carbohydrate metabolism-related enzymes, contributing to the accumulation of carbohydrates (46). Fe sprays increase the concentrations in leaves and decrease the fruit firmness and total phenolic compounds (40, 47), while they are considered a highly effective method for preventing and amending iron chlorosis (48). Although the impact of exogenous mineral treatments on fruits has been studied and utilized extensively, there is still a lack of research focusing on mineral nutrition. Mineral resources of metal salts, which could increase mineral nutrients in fruits, are restricted by oxidation in general use. Fertilizers consisting of the individual metal complex are expected to be more effective than the ones based on unchelated metal salts due to their improved stability and biocompatibility endowed by organic ligands. However, few studies have focused on the effect of poly-metal complex fertilizers on plant growth or fruit quality, successfully improving the contents of various mineral nutrients in fruits through surface treatment remains a challenge.

Given that the total area harvested worldwide of apple exceeds 4.6 mha, with a production of over 86.4 million tons (49), an ideal metallic complex designed to improve the nutrient content of apples can significantly enhance cultivation efficiency and subsequently increase economic value and stimulate the vitality of the market (50). Furthermore, the development and application of an innovative fertilizer with improved stability and reduced cost are desired in agriculture fields. Herein, by fusion of synthetic organic chemistry and plant physiology, we reported the synthetic method of MRP-based antioxidative complexes of Ca, Fe, Zn, and the trimetallic complex and investigated the effects of the complexes on apple mineral nutrients by exogenous treatments. This study provides a novel strategy for integrating metals into one complex. It will serve as a basis for developing effective fertilizers for improving the content of fruit mineral nutrients.

## Materials and Methods

### Materials

Glucose and L-lysine were purchased from Macklin Co., Ltd (Shanghai, China). Ferrous sulfate heptahydrate (FeSO_4_·7H_2_O), calcium chloride (CaCl_2_), and zinc sulfate heptahydrate (ZnSO_4_·7H_2_O) were purchased from the Aladdin Co., Ltd (Shanghai, China). Ethanol, *n*-hexane, and ammonium hydroxide (20%, NH_4_OH) were purchased from Sinopharm Chemical Reagent Co., Ltd (Shanghai, China). Tripyridyltriazine (TPTZ), 1,1-diphenyl-2-picryl-hydrazil (DPPH), and 2,2′-azinobis(3-ethylbenothiazoline-6-sulphonic acid) diammonium salt (ABTS) were obtained from Solarbio Science and Technology Co., Ltd (Beijing, China). Other chemicals were of analytical grade and supplied by Zhongxingweiye Chemicals Co., Ltd (Beijing, China). All chemicals were used as received. Distilled water was self-made.

### Synthesis of Metallic Complexes

Metallic complexes were prepared by reacting metal inorganic salts with glucose and lysine under a thermal process. In brief, to prepare Ca complex (MRPs–Ca), a solution of lysine (7.3 g, 50 mmol) in distilled water (150 ml) was heated to 50°C, followed by a solution of glucose (9.0 g, 50 mmol, 100 ml) was added. After given reaction time at 90°C, CaCl_2_ (5.0 g, 45 mmol) was added to the solution. After 6 h stirring, the solution was cooled down to room temperature, then NH_4_OH (5 ml) was added to precipitate unreacted Ca. Prior to filtration, the solution was kept stirring until NH_4_OH was volatilized completely. The filtrate was freeze-dried and then washed with the solution of ethanol and *n*-hexane (100 ml, *V*_ethanol_:*V*__*n*_−hexane_ =3:1) for 3 times. The final product was obtained after filtration and desiccation under 50°C for 12 h. Fe complex (MRPs–Fe) and Zn complex (MRPs–Zn) were prepared following the method with FeSO_4_·7H_2_O (10.0 g, 40 mmol) and ZnSO_4_·7H_2_O (12.0 g, 42 mmol), respectively. The trimetallic complex (MRPs–Ca/Fe/Zn) was prepared by adding FeSO_4_·7H_2_O (10.0 g, 40 mmol), ZnSO_4_·7H_2_O (12.0 g, 42 mmol), and CaCl_2_ (5.0 g, 45 mmol) into the Maillard reaction system at optimized periods (discussed later). The MRPs were prepared without metal salts.

### Measurement of Reaction Degrees

The reaction degree of glucose-lysine interaction was monitored by the absorbance of the solutions at a wavelength λ of 420 nm. UV spectra were recorded using a Purkinje General TU-1901 spectrophotometer.

### Characterization of Metallic Complexes

The metal contents of the complexes were measured by inductively coupled plasma optical emission spectrometry (ICP-OES) at 25 ± 0.1°C using an Agilent 5110. The size and morphology of the complexes were characterized by transmission electron microscopy (TEM) using an FEI Tecnai G2 F20. The size distribution was determined by measuring at least 50 microspheres from TEM images followed by statistical treatment. The microstructures of the samples were observed by scanning electron microscopy (SEM) with a SEU8010. The zeta potentials of the complexes were measured using a Zetasizer nano. Samples were mixed in absolute ethanol with a concentration of 0.1% (w/v) and immediately transferred into the quartz cuvette for determination. Fourier transform infrared (FTIR) spectra were recorded in the range of 400–4,000 cm^−1^ using a Perkin-Elmer Spectrum 400 spectrometer. The powder samples were dried and ground with potassium bromide in a volume ratio of 1:100. The elemental composition and chemical status of the electrodes were analyzed by X-ray photoelectron spectroscopy (XPS, EscaLab 250, Thermo Fisher Scientific) with a monochromatic Al Kα as the excitation source. The energy calibration was made against the C 1s peak. Thermogravimetric (TG) analyses were performed with a NETZSCH STA 449F3 at the heating rate of 10°C·min^−1^ from 30 to 500°C in a dynamic nitrogen atmosphere.

### Measurement of Antioxidative Activit*y*

The antioxidant capacity by ferric reducing antioxidant power (FRAP) assay is based on the reduction of Fe(III)-tripyridyltriazine (TPTZ) to a blue-colored Fe(II)-TPTZ. The FRAP reagent was prepared with 2.5 ml of 20 mM FeCl_3_·6H_2_O, 25 ml 0.3 mM acetate buffer, and 2.5 ml of a 10 mM 2,4,6-tripyridyl-s-triazine (TPTZ) solution in 40 mM HCl. Vitamin C was used as a standard, the FRAP reagent was used as blank control, and the sample was prepared at a concentration of 1 mg·ml^−1^ (Supplementary Figure 1). Notably, 30 μl of the sample with 5-fold dilution, standard, and blank reagent were mixed with 500 μl of FRAP reagent and 2,000 μl of distilled water, respectively. After 30 min incubation at 37°C, the absorbance of the solutions at a wavelength λ of 593 nm was recorded using a Purkinje General TU-1901 spectrophotometer. Calibration was performed with vitamin C solution.


(1) $$\begin{array}{r} \text{Ferric reducing antioxidant power \%}\\ \;=\;\left(\frac{A_{\text{sample}}\;-\;A_{\text{control}}}{A_{\text{sample}}}\right)100\% \end{array}$$

The radical scavenging activity was determined by the decolorization of 1,1-diphenyl-2-picryl-hydrazil (DPPH). The DPPH regent was prepared at a concentration of 0.12 mM and protected from light. Vitamin C was used as a standard, the DPPH reagent was used as blank control, and the sample was prepared at a concentration of 1 mg·ml^−1^ (Supplementary Figure 1). An aliquot of 1 ml of DPPH reagent was added to 100 μl of the sample with 5-fold dilution and 2 ml of distilled water. The solution was allowed to stand at room temperature in the dark for 30 min. The absorbance of the solutions at a wavelength λ of 515 nm was recorded using a Purkinje General TU-1901 spectrophotometer. The antiradical activity was expressed as a percentage of the disappearance of the initial purple color. Calibration was performed with vitamin C solution.


(2) $$\begin{array}{r} \text{DPPH radical scavenging activity \%}\\ \;=\;\left(\frac{A_{control}\;-\;A_{sample}}{A_{control}}\right)100\% \end{array}$$

The radical cation decolorization activity was determined based on 2,2′-azinobis(3-ethylbenothiazoline-6-sulphonic acid) diammonium salt (ABTS). The ABTS regent was prepared at a concentration of 7 mM with 2.45 mM potassium persulfate and kept in the dark at room temperature for 12 h. Vitamin C was used as a standard, the DPPH reagent was used as blank control, and the sample was prepared at a concentration of 1 mg·ml^−1^ (Supplementary Figure 1). An aliquot of 1 ml of the solution was mixed with 30 μl of the sample and 2 ml of distilled water for 45 s. The absorbance of the solutions at a wavelength λ of 734 nm was recorded using a Purkinje General TU-1901 spectrophotometer. The antiradical activity was expressed as the percentage of the disappearance of the initial blue color. Calibration was performed with vitamin C solution.


(3) $$\begin{array}{r} \text{ABTS radical scavenging activity \%}\\ \;=\;\left(\frac{A_{control}\;-\;A_{sample}}{A_{control}}\right)100\% \end{array}$$

### Plant Materials

The spraying experiment was conducted in an orchard located in Beijing, China (39° 57′ 59″ N, 116°13′ 22″ E). Six-year-old trees of apple (*Malus domestica* cultivars “Fuji”) were chosen based on the uniformity of tree size. Trees were not treated with growth regulators or phytohormone-containing preparations. Control of pathogens and pests was performed according to the recommendations for integrated apple production.

### Treatments and Experiment Layout

The concentration of solution for spray was based on the mass fraction of the metal element. The control treatment was sprayed with water. Low (L), medium (M), and high (H) concentrations of MRPs–Ca/Fe/Zn were sprayed at a given period (Table 1). For uniform coverage, 10 L solution was applied per tree, 0.2% tween-20 (v/v) was added to the solutions as a surfactant. The leaves and fruits without any covers were sprayed on the surfaces with machines backpack up to runoff in the morning (8:00–10:00 a.m.). Each treatment consisted of 3 replicates with 3 trees. Ten fruit samples were randomly harvested from each tree. Leaf samples were collected from the middle part of the new branches of the year.

**Table 1:** The design of the spray treatment of MRPs- Ca/Fe/Zn.

**Treatment**	**Concentration^a^**	**Period**				
		**May 15th**	**June 15^**th**^**	**July 16th**	**August 15th**	**December 17th**
T1	L	√				√
T2	L	√		√		√
T3	L	√	√	√	√	√
T4	M	√				√
T5	M	√		√		√
T6	M	√	√	√	√	√
T7	H	√				√
T8	H	√		√		√
T9	H	√	√	√	√	√
Control^b^		√	√	√	√	√

a *The concentration was calculated by mass fraction of the metal element. L (Ca: 0.27%, Fe: 0.25%, and Zn: 0.29%); M (Ca: 0.54%, Fe: 0.5%, and Zn: 0.58%); H (Ca: 0.9%, Fe: 0.83%, and Zn: 0.97%)*.

b *The control was performed by distilled water*.

### Determination of Fruit Quality

To measure the weight (g), the fruits immediately after harvest were recorded using a digital balance. Flesh firmness was determined using a force gauge (GY-1, WDGage, China) on the two opposite sides of the fruit without skin.

To evaluate fruit soluble solid contents (SSCs) and titratable acidity (TA), a sample of juice was taken from one piece of each fruit. SSCs were determined using a digital refractometer (PAL-BX/ACID12, Atago, Japan) and expressed as a percentage. TA was determined by titrating 3 ml of juice in 27 ml distilled H_2_O with 0.1 N NaOH to pH 8.1 and expressed as % malic acid.

To assess the mineral composition, 5 g leaves were washed with distilled water, and 5 g peels and 10 g longitudinal slices were obtained from each fruit, respectively. The samples were dried in an oven at 105°C for 30 min for deactivation of enzymes and then at 75°C for 48 h for dehydration. Ash was produced at 500°C for 8 h, then dissolved in 5 ml of 1N HCl, and filtered through a filter paper. Concentrations of Ca, Fe, and Zn were determined using an atomic absorption spectrophotometer (Z-2000, Hitachi, Japan) at 422.7, 248.3, and 213.9 nm, respectively.

To determine the total N content, the Kjeldahl method was used. The content of N was determined using a continuous flow analysis system (AA3, SEAL, Germany) at 660 nm.

### Statistical Analysis

Statistical analysis was performed using SPSS (version 11.0, SPSS Inc., Chicago, USA). The data were subjected to the analysis of variance and expressed as the mean ± standard error (SE) (*n* = 3). Mean comparisons were performed using the least significant difference (LSD) test at a significance level of *P* < 0.05.

## Results and Discussion

### Synthesis of the Trimetallic Complexes

**Figure 1:**
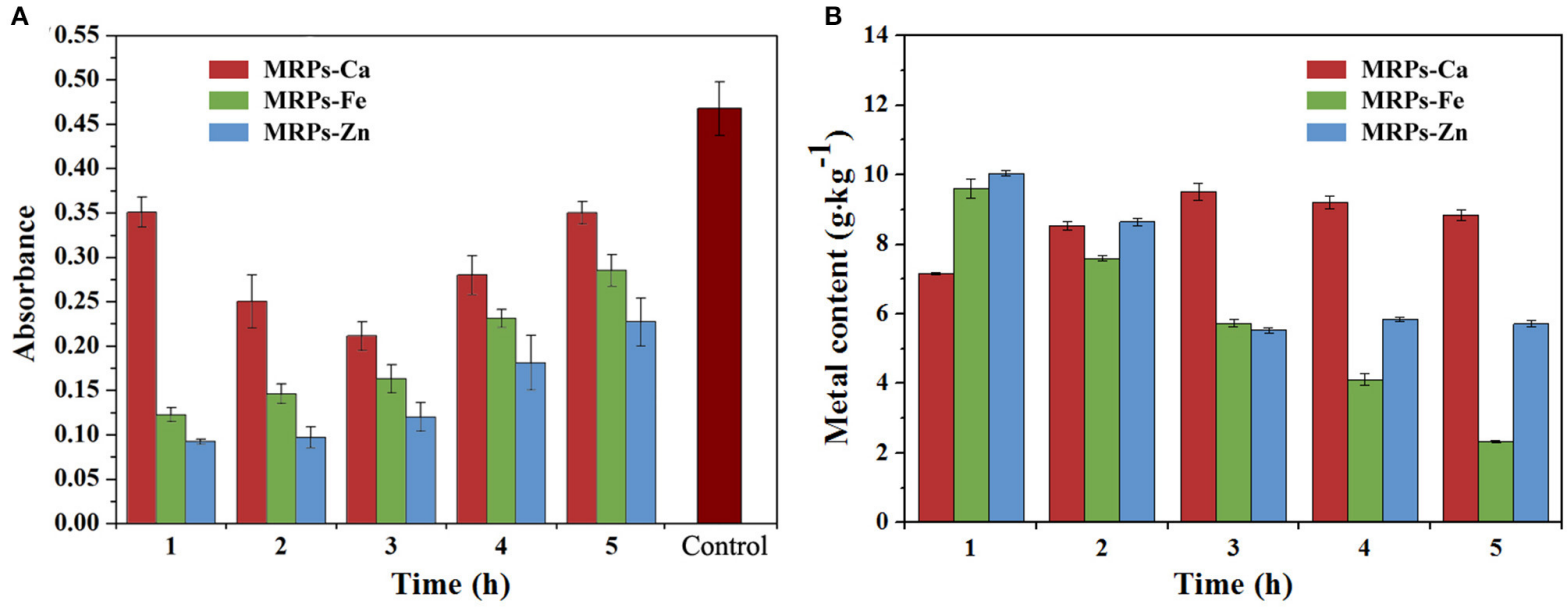
Reaction degrees of the Maillard reaction (MR) **(A)** and metal contents of Maillard reaction products (MRPs)–Ca, MRPs–Fe, and MRPs–Zn **(B)**. The horizontal axis presents the reaction time of the MR when the metal is added. The control is MR without metals.

The complexes were synthesized under heating conditions in the presence of glucose and lysine, a classical MR with high reactivity (51). To obtain high coordination ability with metal ions, a time-based synthetic process was studied. Superfluous metal salts were added into the system at different periods of the MR system, and metal combination rates were determined by checking the mass percentages of the final products. Prior to that, ammonia water was used to remove unreacted metals. Since the reason for the interactions of metals and MRPs was different, the reaction process and coordination were different. As shown in Figure 1, the introduction of metals depressed the degree of MR. In the presence of metals, the degree increased when Ca^2+^ was added at an earlier or later stage; however, there was no significant change in the Ca content of MRPs–Ca whenever Ca^2+^ was added into the system (Supplementary Table 1). In contrast, premature addition of Fe^2+^ and Zn^2+^ caused a lower degree of MR, suggesting that Fe^2+^ and Zn^2+^ have inhibitory effects on the MR. However, higher Fe and Zn contents of MRPs–Fe and MRPs–Zn were achieved by adding inorganic salts in the initial periods. Therefore, to load more metals, the MRPs–Ca, MRPs–Fe, and MRPs–Zn were synthesized by adding the relevant salts after 3, 1, and 1 h of MR, respectively. The addition of Ca^2+^ and ${\text{SO}}_{4}^{2-}$ in the system at different periods helped prepare soluble substances. Ultimately, MRPs–Ca/Fe/Zn was synthesized with 9.01%, 8.25%, and 9.67% contents of Ca, Fe, and Zn, respectively.

### Structure of the Trimetallic Complexes

**Figure 2:**
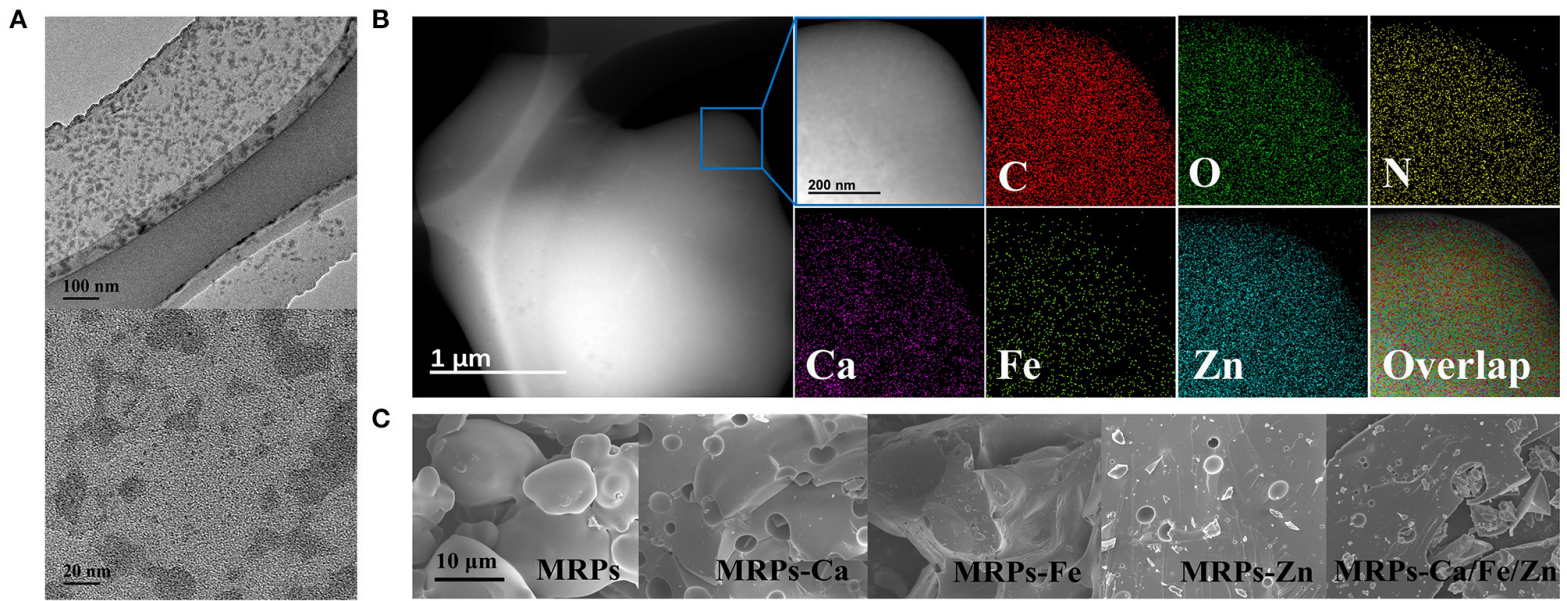
Transmission electron microscopy (TEM) images of MRPs–Ca/Fe/Zn **(A)**. STEM images and corresponding elemental mapping analysis of MRPs–Ca/Fe/Zn **(B)**. Scanning electron microscopy (SEM) images of MRPs, MRPs–Ca, MRPs–Fe, MRPs–Zn, and MRPs–Ca/Fe/Zn **(C)**.

The TEM images (Figure 2A) illustrate that MRPs–Ca/Fe/Zn was amorphous owing to the glycosylated MRP structure. Metal ions were dispersed on film-like MRPs with partial aggregation of <30 nm. The elemental mapping images clearly demonstrate that Ca, Fe, and Zn were uniformly distributed in MRPs–Ca/Fe/Zn, indicating that the three metals were successfully integrated into the MRPs (Figure 2B). The SEM images (Figure 2C and Supplementary Figure 2) show that the morphology of the MRPs turned from a quasi-circular spheroid with a smooth surface into a blocky structure with non-homogeneous defects by interacting with the metals. The ζ-potential of the MRPs changed from 0.02 to −4.06, 1.38, 3.11, and 1.30 for the MRP-based Ca, Fe, Zn, and Ca/Fe/Zn complexes, respectively (Supplementary Figure 3 and Supplementary Table 2).

**Figure 3:**
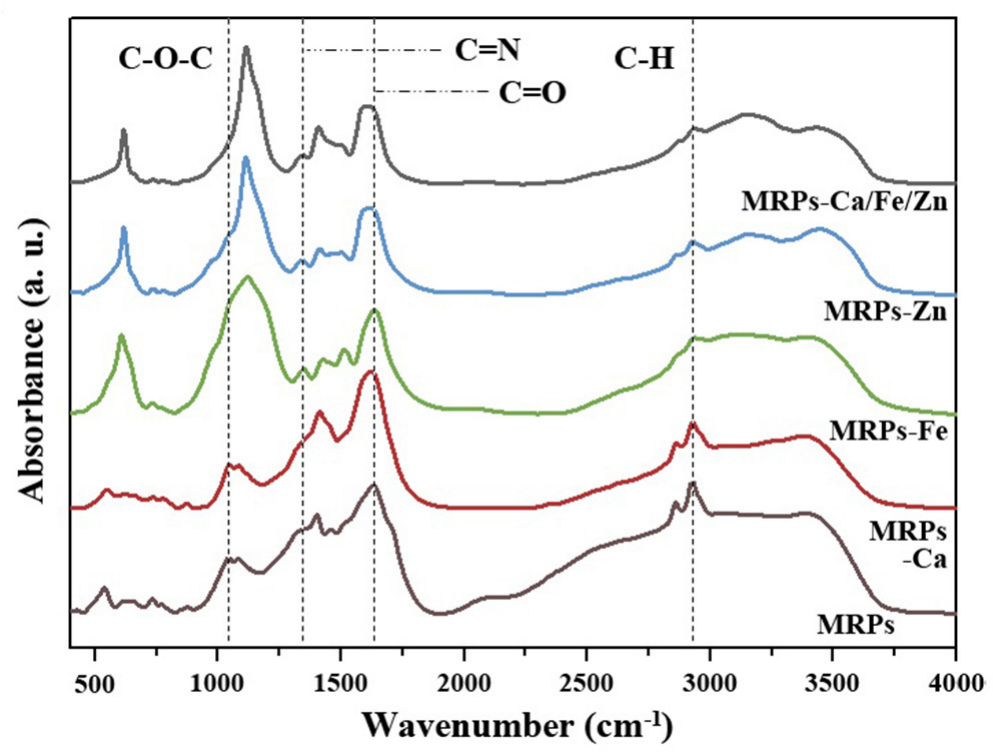
Fourier transform infrared (FTIR) spectra of MRPs, MRPs–Ca, MRPs–Fe, MRPs–Zn, and MRPs–Ca/Fe/Zn.

The FTIR spectra of MRPs–Ca, MRPs–Fe, MRPs–Zn, and MRPs–Ca/Fe/Zn significantly differed from the raw materials (Supplementary Figures 4, 5), indicating the formation of the complexes. Figure 3 shows that all complexes contained huge amounts of –OH groups and intermolecular hydrogen bonds based on the broad peaks observed at 3,000–3,600 cm^−1^. Moreover, the spectra suggest that the complexes contain crystalline water. The peak of the C–H vibration centered at 2,927 cm^−1^ of the MRPs was weakened by interaction with metal ions. The peak at 1,629 cm^−1^ of the MRPs, which could be regarded as the C=O of the carboxyl group, was red-shifted in MRPs–Ca, MRPs–Zn, and MRPs–Ca/Fe/Zn, indicating the formation of O–metal bonds (52). The similarity in the spectra of MRPs–Ca and MRPs was caused by the equal reaction degrees, which correspond to the insignificant influence of Ca^2+^ on the MR. The peaks at 607 and 1,121 cm^−1^ in MRPs–Fe, MRPs–Zn, and MRPs–Ca/Fe/Zn, attributable to ${\text{SO}}_{4}^{2-}$, were prominent, making the peaks of the C–O–C of the aromatic alkoxy at ~1,042 cm^−1^ to appear as shoulders (53). The coexistence of Ca^2+^ and ${\text{SO}}_{4}^{2-}$ confirmed that the metal was prevented from precipitating. Consequently, the interaction of metal ions and MRPs was confirmed, and the trimetallic complex was successfully obtained.

**Figure 4:**
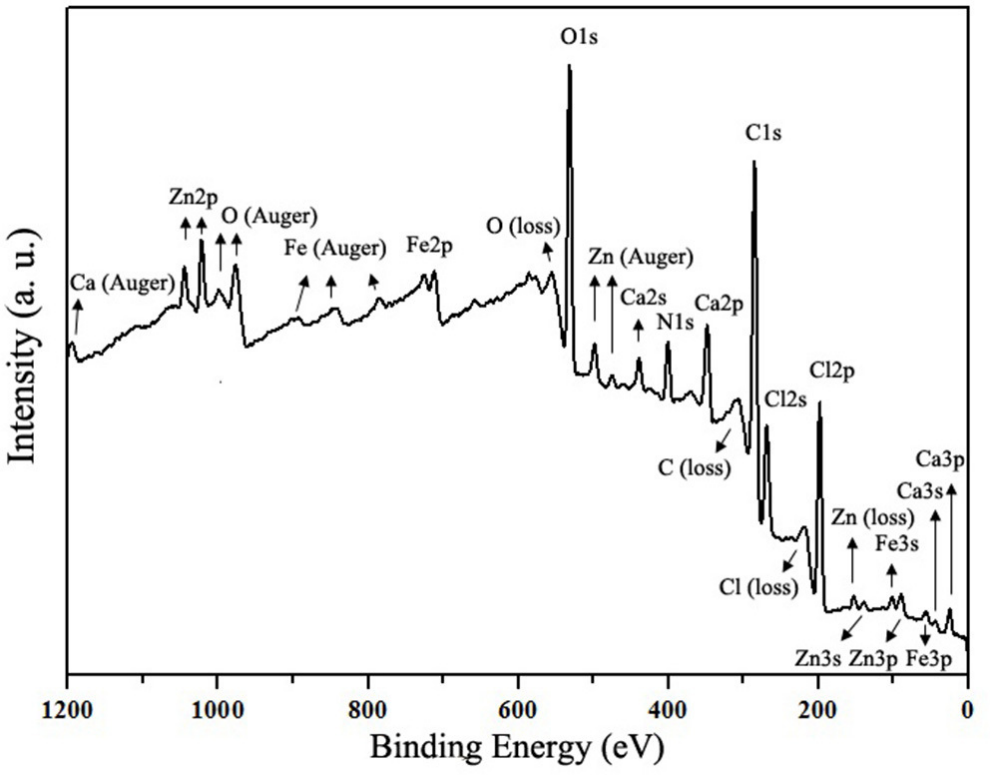
X-ray photoelectron spectroscopy (XPS) spectrum of MRPs–Ca/Fe/Zn.

**Figure 5:**
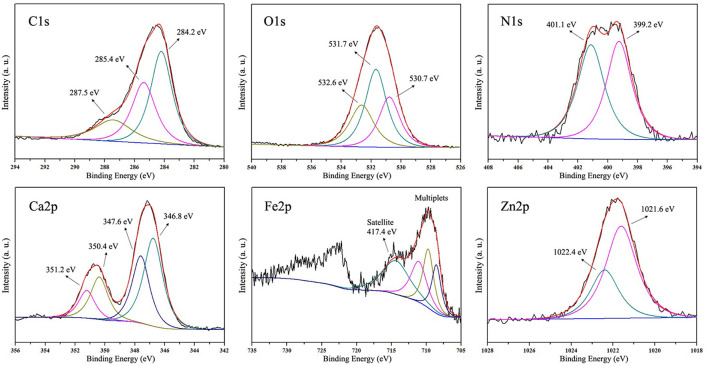
The deconvoluted high-resolution XPS spectra of MRPs–Ca/Fe/Zn.

The full-scan XPS spectrum of MRPs–Ca/Fe/Zn is shown in Figure 4. The complex contained C, O, N, Ca, Fe, Zn, and Cl. The prominent peaks of C 1s and O 1s at a binding energy of ~285 and 532 eV, respectively, were originated from the MRPs on the surface. Besides, the intensities of photoelectron lines of Cl were relatively higher, indicating the Cl^−^ preferred to combine on the surface. The deconvoluted high-resolution XPS spectra are illustrated in Figure 5. The three C 1s peaks at 284.2, 285.4, and 287.5 eV were assigned to the carbon as C–C/C–H, C–O/C–N, and C=O, respectively. The deconvoluted O 1s was fitted with three components at 530.7, 531.7, and 532.6 eV belonging to metal–O, O–H, and surf–O/C–O, respectively (24, 54). The N 1s peak can be deconvoluted into two peaks at 399.2 and 401.1 eV, which assigned, respectively, to pyridinic-N/metal–N and C–N/N–H (24, 55). The spectrum of Ca shows the 2p_3/2_ and 2p_1/2_ at 347.1 and 350.6 eV, respectively, and the binding energies of Ca 2p_3/2_ peak can be deconvoluted into 346.8 and 347.6 eV, suggesting the interaction between COO^−^ and Ca^2+^ (56). The peaks of Fe 2p_3/2_ and 2p_1/2_ were split into independent satellite peaks due to the major component of Fe(II), indicating that the Fe^2+^ were prevented from oxidation by the MRPs (57). The peak of Zn 2p_3/2_ at 1,021.8 eV was deconvoluted into 1,021.6 and 1,022.4 eV, which were, respectively, assigned to Zn–O and Zn–Cl (54, 58). This revealed that ZnCl_2_ was generated due to the metathetical reaction in the presence of CaCl_2_, thus ZnCl_2_ was recommended for the preparation of MRPs–Ca/Fe/Zn.

### Thermal Stability of the Trimetallic Complexes

**Figure 6:**
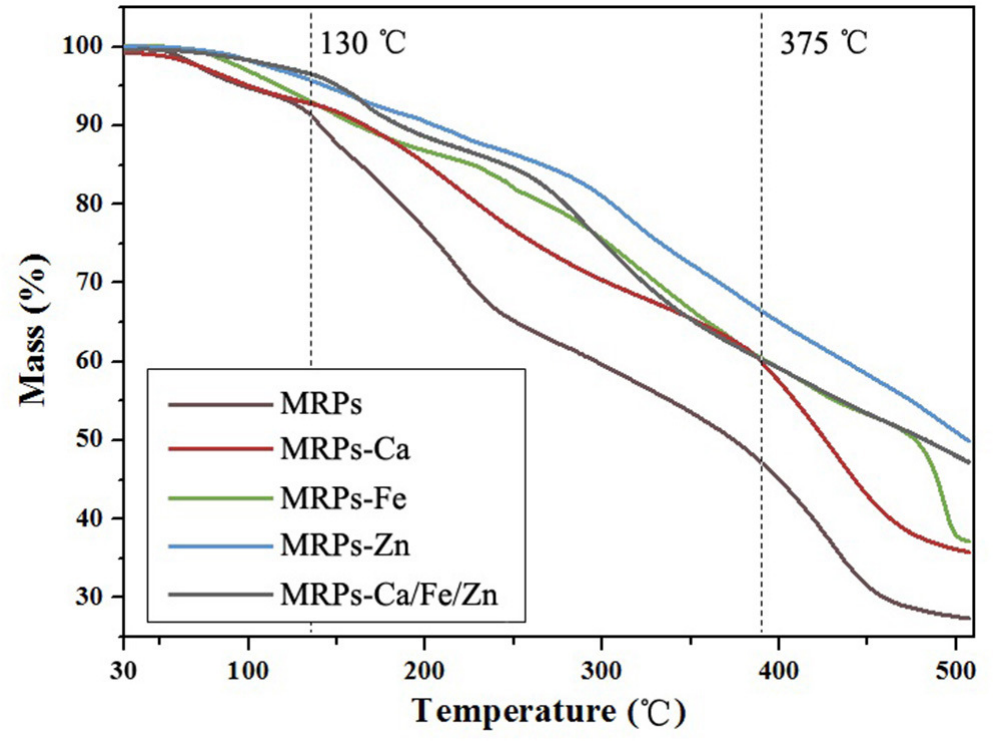
Thermogravimetric (TG) plots of MRPs, MRPs–Ca, MRPs–Fe, MRPs–Zn, and MRPs–Ca/Fe/Zn.

The TG analysis presented in Figure 6 shows the different mass losses of the complexes. The loss below 130°C was attributed to dehydration and dissociation of molecules from the sample surface (59). In practice, the use of glucose and lysine units rendered the complexes hydrophilic and hygroscopic, and careful preservation is recommended. The losses of MRPs, MRPs–Ca, and MRPs–Ca/Fe/Zn accelerated from 130°C, but no obvious acceleration was observed for MRPs–Fe and MRPs–Zn until 251°C and 293°C, respectively. The huge losses incurred from 375°C in MRPs and MRPs–Ca were due to the decomposition of the MRP molecules and release of methane (60); the loss in MRPs–Fe occurred at 472°C (Supplementary Figure 6). However, MRPs–Zn and MRPs–Ca/Fe/Zn underwent an insignificant loss process instead of severe ones. Overall, the complexes are competent in the normal environment of agricultural application.

### Antioxidative Activity of the Trimetallic Complexes

**Figure 7:**
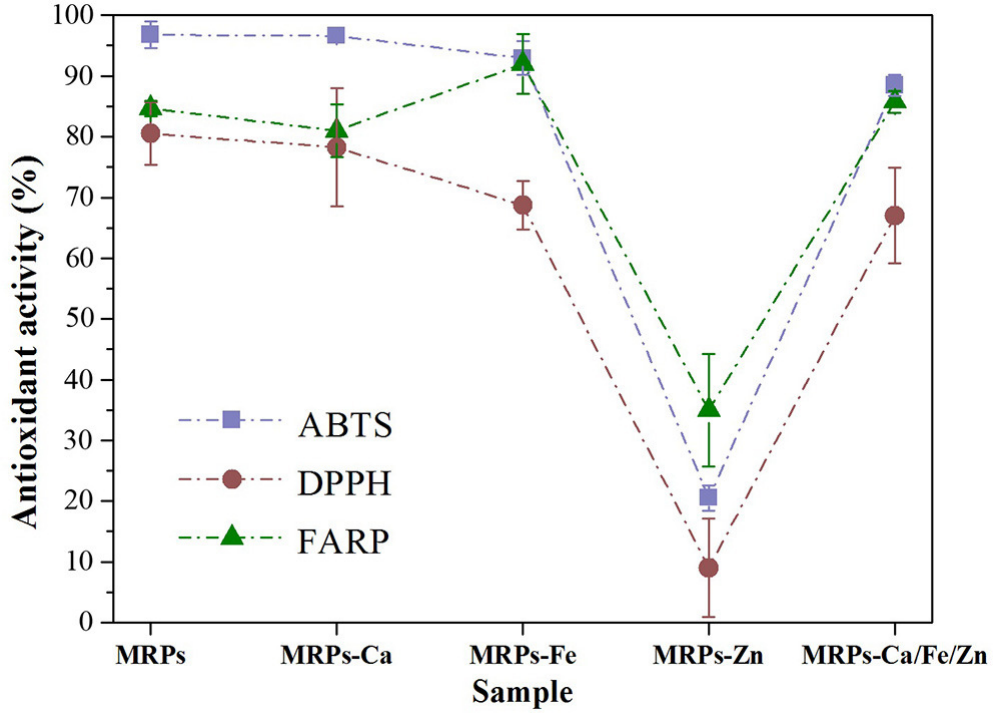
Antioxidative activity analysis of MRPs, MRPs–Ca, MRPs–Fe, MRPs–Zn, and MRPs–Ca/Fe/Zn.

Antioxidant assays were performed to determine the complexes' ability to resist oxidative stress and scavenge reactive oxygen species and thus assess their biocompatibilities. ABTS, DPPH, and FRAP assays exhibited a similar law of antioxidant activities among the metallic complexes. As shown in Figure 7 and Supplementary Table 3, the activities of the MRPs were slightly lower than the standard and decreased on interaction with metal ions. The decreases in MRPs–Fe and MRPs–Zn were due to not only the introduction of metals but also the lower degree of the MR. Fortunately, the sharp loss due to Zn^2+^ could be restored by constructing a trimetallic complex. Distinctively, the activity of MRPs–Fe was higher than that of others in the FRAP assay. This is because the FRAP assay based on the reduction of Fe (III) to Fe (II) relies on the number of ferrous ions captured by TPTZ; a large number of ferrous ions from MRPs–Fe improved the association of TPTZ with Fe (II), resulting in the improved activity of FRAP. Overall, the complexes were endowed with considerable antioxidative activity by MRPs; thus, they have great potential in biological applications.

### Effect of the Trimetallic Complexes on Apple

A field experiment was performed by spraying the solution of the complexes on apple trees with different concentrations and periods (Table 1). With the advantages associated with the complex structures, including high hydrophilicity due to the glycosylated coating and antioxidation due to protection from the MRPs, metal ions become highly stable under aqueous conditions. No oxidation or precipitation occurred during the spray. It is propitious for improving the efficiency of agrochemicals and reducing fertilizer consumption. Pretreatments showed that no damages were observed on the fruit surfaces (Supplementary Figure 7) under the high concentration treatments of MRPs–Ca, MRPs–Fe, MRPs–Zn, and MRP–Ca/Fe/Zn, suggesting that the complexes had certain biocompatibility (61). Given that we are focusing on the poly-metal complex fertilizer, the solutions of MRP–Ca/Fe/Zn were prepared for further experiments.

**Figure 8:**
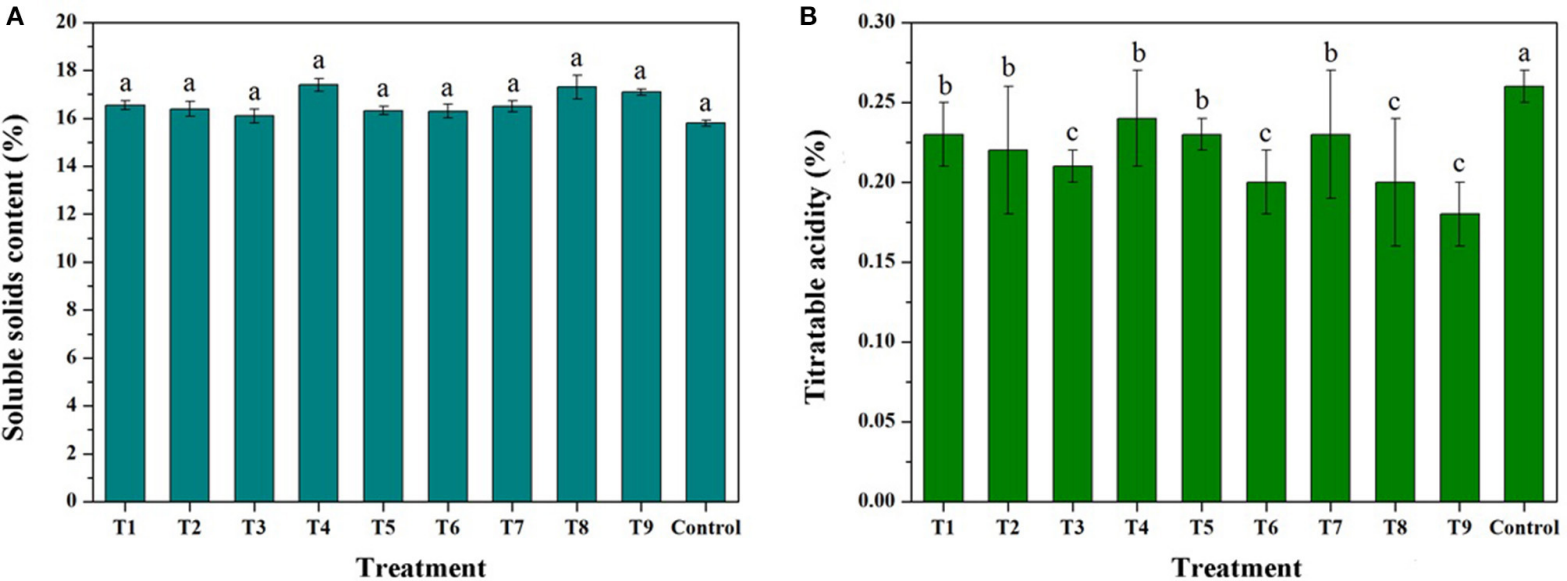
Effects of MRPs–Ca/Fe/Zn on soluble solid contents (SSCs) **(A)** and titratable acidity (TA) **(B)** of the fruits.

Figure 8 and Supplementary Table 4 show the SSCs and TA contents of the treated fruits. The SSCs can be slightly increased by increasing the concentration of the trimetallic complex, but the index decreases with an increase in the number of sprays. It is reported that the spray treatment by CaCl_2_ can result in a reduction in SSCs, even if the Ca content of a mature apple is increased (62). In contrast, foliar treatment with sugar alcohol zinc lead to increased sugar concentration, probably attributed to the significant effect of sugar alcohol zinc on sucrose synthase activity which regulates sugar accumulation (63). Therefore, the effect of MRPs–Ca/Fe/Zn on SSCs could be bidirectional, and excessive treatment would lead to a decrease in the content. For TA, majority treatments can decrease TA contents compared with the control, and the contents decreased with increasing concentration and number of sprays of MRPs–Ca/Fe/Zn. The treatment of T9 decreased the TA content from 0.26 to 0.19%. This trend of change is consistent with the reported studies (64, 65), but it is shown that the single application of amino acid triggers increasing in TA (65), suggesting that the reduction of TA is associated with mineral content. However, it also reported that the SSCs and TA are almost unchanged under the treatment of the chelated-metal or nanochelated-metal (66), indicating that the effect of MRPs–Ca/Fe/Zn was attributable to not only the concentration and period of application but also the complicated metabolic mechanism and environmental conditions (67).

As shown in Supplementary Table 4, the weight and firmness show no obvious regularity and did not differ among the treatments, indicating that MRPs–Ca/Fe/Zn hardly improved the two indices of the “Fuji” apple. It is known that the fruit concentration of Ca is positively correlated with firmness owing to the cell wall resistance that can be elevated by increasing the Ca level (68, 69). The slight decrease of firmness could be the reason that the MRP-based complex affected the fruit starch degradation pattern which has strong negative correlations with fruit firmness (70, 71).

**Figure 9:**
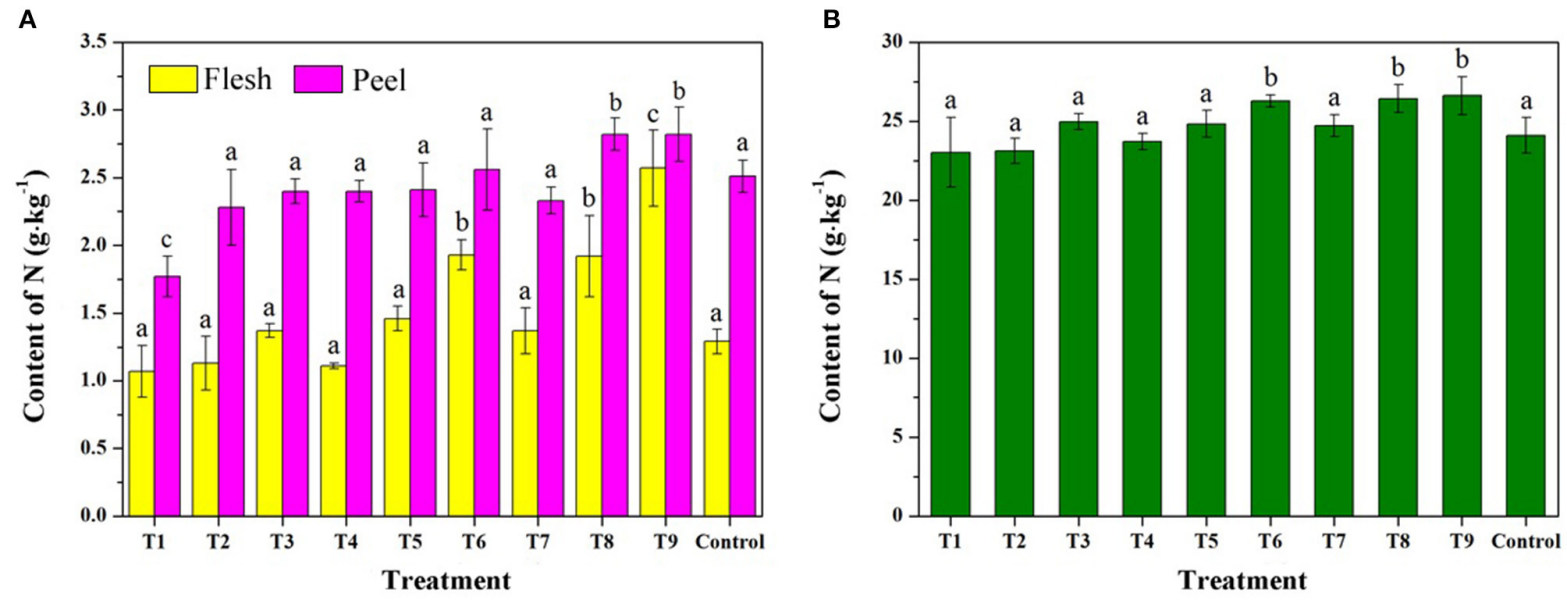
Effects of MRPs–Ca/Fe/Zn on N contents of fleshes **(A)**, peels **(A)**, and leaves **(B)**.

The results of N content analysis are shown in Figure 9 and Supplementary Table 5. The N contents of fleshes, peels, and leaves increased following the order of T1–T9, indicating that the MRPs containing N groups were absorbed into the plants. Compared with the control, MRPs–Ca/Fe/Zn sprays enhanced flesh N concentrations on average by 6.2, 49.6, and 99.2% of T3, T6, and T9, respectively. The growth rates in fleshes were high than that in peels and leaves, indicating that the trimetallic complex can improve the accumulation of N in the fruit flesh. The results were inconsistent with the N contents regulated by inorganic calcium salt (45), demonstrating the promotion effect of MRPs to the accumulation of N content. However, the mechanism remains unclear.

**Figure 10:**
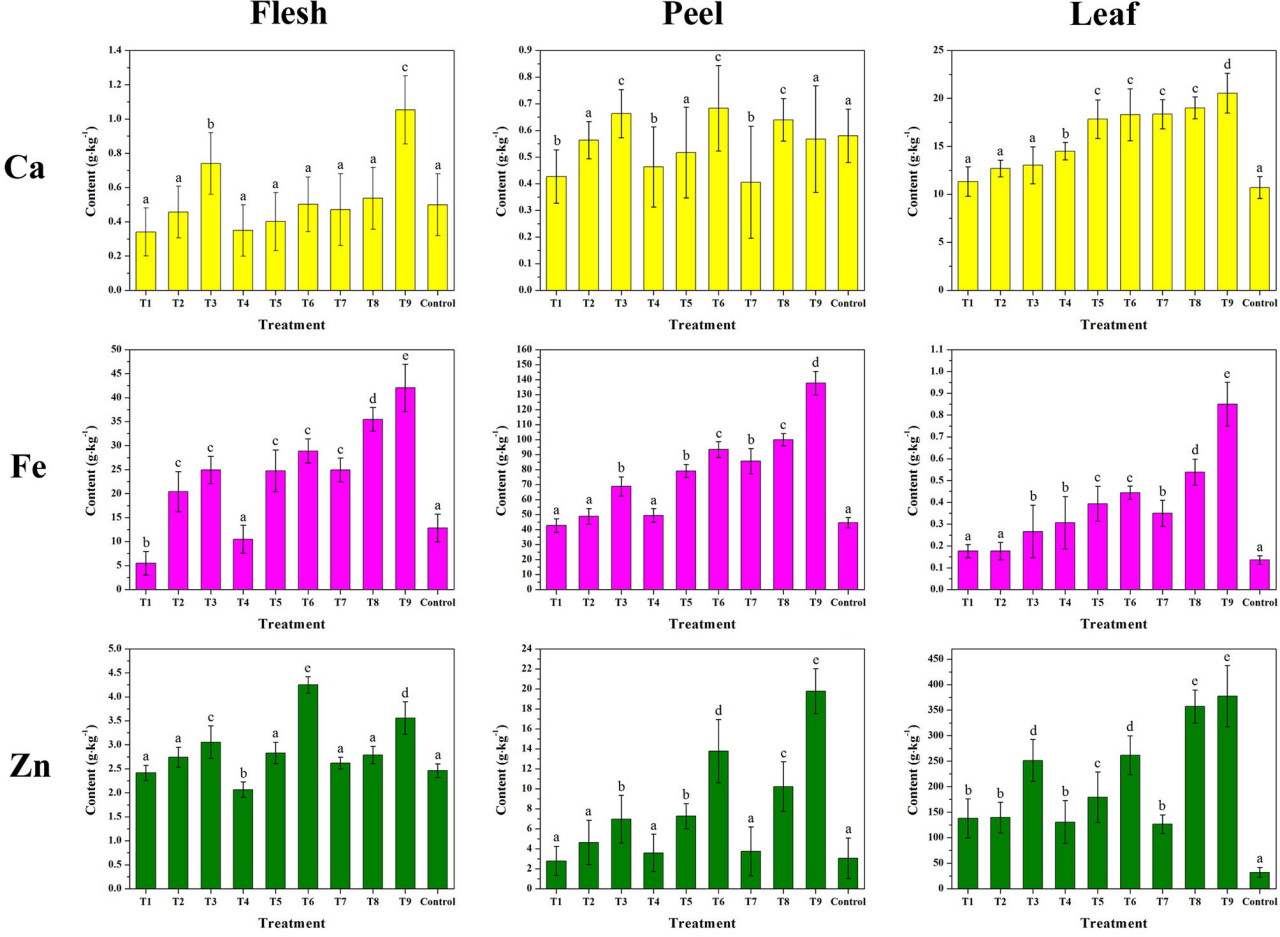
Effects of MRPs–Ca/Fe/Zn on the mineral nutrients of fleshes, peels, and leaves of “Fuji” apple.

Figure 10 and Supplementary Table 6 compare the contents of Ca, Fe, and Zn contents of fleshes, peels, and leaves of the harvested fruit. The results were in agreement with the mineral spray treatment taken on pear (47), grape (40), pomegranate (3), and orange (72). Overall, the metal contents increased with the increasing concentration and treating time of the complexes. Compared with the control, the enhanced mineral contents of T1–T9 were attributed to the additional metal absorbed into the plant by the MRPs. The rise of the mineral contents in leaves was higher than the other two parts owing to the higher rate of water supply and nutrient transport than the rest of the plant (73). Although the report shows that increasing Fe rates decreased leaf Zn contents due to the internal disturbance in the nutritional balance (41), the MRPs–Ca/Fe/Zn could drive the two contents' growth simultaneously. The rises in Fe and Zn contents in the peel were higher than those in the flesh, indicating that most of the two minerals were detained in the peel. However, the reverse order of Ca suggests that Ca passed through the skin and into the flesh with enhanced delivery efficiency. According to previous studies (45, 74, 75), Ca lacks the effectiveness to penetrate the fruit epidermis, the spraying of CaCl_2_ or CaCO_3_ hardly affects the concentration of flesh, and several treatments are needed to increase the concentration of Ca in the peel. Therefore, considering the results in this study, MRPs could improve the mobility and transportation of Ca to penetrate the plant cell. However, an insufficient amount of MRPs–Ca/Fe/Zn (T1, T4, T5, and T7) could decrease the Ca concentration in fleshes, as well as in peels, it implies that the combined effect of polymetallic foliar fertilizer might reduce the absorb efficiency of calcium, thus increase in dosage is recommended.

Notably, T9 of MRPs–Ca/Fe/Zn (Ca: 9.01%, Fe: 8.25%, and Zn: 9.67%) provided the following increments in Ca, Fe, and Zn contents: 85.4, 532.5, and 931.1% in the leaf; 16.0, 225.2, and 468.6% in the peel; and 117.6, 217.9, and 19.5% in the flesh, respectively. Therefore, the increase in mineral content did not directly relate to the metal content of the complex. Although the impact of the treatment on Ca in peel and Zn in flesh is subtle, none of the nutritional concentrations were negatively altered by the treatment relative to the control. Higher increments correlated with the efficiencies of absorption and metabolism; the modified metal provided more nutrients for these processes. Both sugar- and amino-based metallic materials have better efficiency for promoting the mineral absorption of apple than inorganic salts (62, 63, 76). It is noteworthy relative to the unchelated metals, chelated metal complex with improved stability can be conducive to mineral biofortification in fruits, although the mechanism is still unclear. Remarkably, the MRP-based complexes, in which the chelated ligands were derived from the reaction of sugar and amino acid, could lead to a higher growth rate of the mineral content in apples. These results indicate that the antioxidative ligands significantly contributed to the process of Ca, Fe, and Zn uptake by plants. Therefore, although the improvements in Ca, Fe, and Zn by MRPs–Ca/Fe/Zn were not identical, the bio-utilization of minerals would profit from the modified metals, which are coordinated with glycosylated compounds, resulting in uniform dispersion at minimal scale while being transported into the plant at highly active rates.

An identical treatment was carried out in the next year to test and verify the reliability and reproducibility of the method by introducing only the T9 in view of the highest efficiency. The effect on Ca, Fe, and Zn of the samples from 2020 to 2021 is compared in Table 2. Although the contents of Ca in flesh, Zn and Fe in the peel, and Zn in leaf were lower than those in 2020, these data did not differ significantly. The reason for the discrepancy might be due to the differences in plant growth conditions (soil and/or environmental) of the 2 years. Taking into account the data in the 2 years, we can attribute that this method for improving mineral concentration in cultivar apple is effective and reproductive.

**Table 2:** Content of mineral nutrients of the “Fuji” samples in 2 years.

**Content (g·kg^**−1**^)**	**Flesh**		**Peel**		**Leaf**	
	**2020**	**2021**	**2020**	**2021**	**2020**	**2021**
Ca	1.05 ± 0.2	0.97 ± 0.15	0.57 ± 0.23	0.73 ± 0.63	20.54 ± 2.08	22.13 ± 2.74
Fe	42.06 ± 4.95	55.65 ± 3.88	137.72 ± 7.85	124.53 ± 3.44	0.85 ± 0.1	1.12 ± 0.31
Zn	3.56 ± 0.34	4.12 ± 1.14	19.78 ± 2.26	16.85 ± 2.09	377.59 ± 59.8	356.46 ± 36.76

## Conclusion

We developed a new approach to integrate Ca, Fe, and Zn with the biodegradable ligand which is derived from the thermal reaction of saccharide and amino acids. The metals are evenly distributed over the ligands with insignificant aggregation. The resultant complexes possess desirable stability and antioxidation to prevent metals from oxidizing, thereby enhancing stability under aqueous conditions and mineral transport efficiency into plants. The biological assay showed that mineral nutrients of Ca, Fe, and Zn in apple simultaneously and remarkably increased through surface spraying of the metal–integrated complex, and the efficiency is much higher than the traditional inorganic salts and single chelated metals. The proposed method will inspire new insights and methodologies to develop green agricultural technology.

## Data Availability Statement

The original contributions presented in the study are included in the article/Supplementary Material, further inquiries can be directed to the corresponding author.

## Author Contributions

Y-ZY: writing—original draft, formal analysis, methodology, investigation, and funding acquisition. Q-PW: conceptualization, supervision, and funding acquisition. JZ: data curation, investigation, visualization, and writing—review and editing. M-JL and B-BZ: writing—review and editing. QZ: investigation and writing—review and editing. X-LL: supervision and visualization. J-KZ: supervision, project administration, and writing—review and editing. All authors contributed to the article and approved the submitted version.

## Funding

This study was supported by the National Key Research and Development Program of China (No. 2020YFD1000201), the National Natural Science Foundation of China (No. 22005037), and the Agriculture Research System of China (CARS-27) for the funding.

## Conflict of Interest

The authors declare that the research was conducted in the absence of any commercial or financial relationships that could be construed as a potential conflict of interest.

## Publisher's Note

All claims expressed in this article are solely those of the authors and do not necessarily represent those of their affiliated organizations, or those of the publisher, the editors and the reviewers. Any product that may be evaluated in this article, or claim that may be made by its manufacturer, is not guaranteed or endorsed by the publisher.
