# Supplementary material for: Nano-Sized Antioxidative Trimetallic Complex Based on Maillard Reaction Improves the Mineral Nutrients of Apple (Malus domestica Borkh.)
Source: Front Nutr. 2022 Apr 25;9:848857. doi: 10.3389/fnut.2022.848857 (PMC9086434; doi:10.3389/fnut.2022.848857)
Supplement: Supplementary file 1 [file Data_Sheet_1.docx]

Nano-sized Antioxidative Trimetallic Complex Based on Maillard Reaction Improves the Mineral Nutrients of Apple (*Malus domestica* Borkh.)

**Yu-zhang Yang^1^, Qin-ping Wei^1^, Jia Zhou^1^, Min-ji Li^1^, Qiang Zhang^1^, Xing-liang Li^1^, Bei-bei Zhou^1^, Jun-ke Zhang^1*^**

^1^Institute of Forestry and Pomology, Beijing Academy of Agriculture and Forestry Sciences, Beijing, China


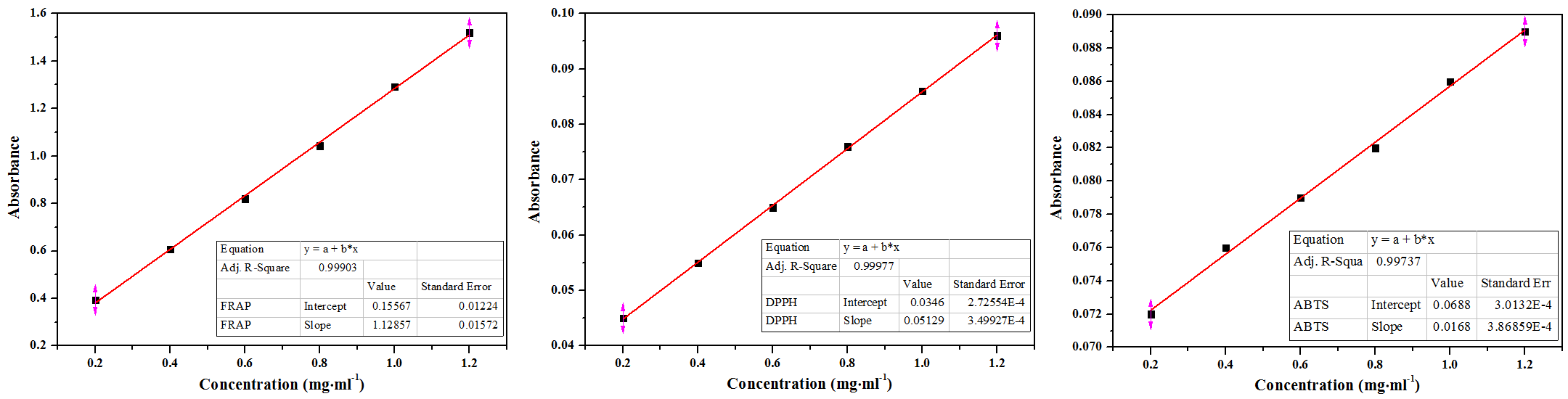


**Figure S1.** Antioxidation ability of vitamin C.

**Table S1.** Metal contents of the complexes and reaction degrees of the MR in different reaction processes.

| Time (h)^a,b^ | Content^c^ (g·kg^-1^) | | |  | Absorbance^d^ | | |
| --- | --- | --- | --- | --- | --- | --- | --- |
|  | MRPs-Ca | MRPs-Fe | MRPs-Zn |  | MRPs-Ca | MRPs-Fe | MRPs-Zn |
| 1 | 7.15±0.03 | 9.61±0.027 | 10.04±0.07 |  | 0.351±0.017 | 0.1225±0.008 | 0.092±0.003 |
| 2 | 8.53±0.13 | 7.59±0.08 | 8.64±0.1 |  | 0.25±0.03 | 0.146±0.011 | 0.097±0.012 |
| 3 | 9.51±0.25 | 5.73±0.1 | 5.53±0.07 |  | 0.211±0.016 | 0.163±0.016 | 0.12±0.016 |
| 4 | 9.2±0.18 | 4.11±0.16 | 5.84±0.06 |  | 0.28±0.022 | 0.231±0.01 | 0.181±0.031 |
| 5 | 8.84±0.14 | 2.33±0.03 | 5.72±0.08 |  | 0.35±0.013 | 0.285±0.018 | 0.227±0.027 |

a It indicated the reaction time of glucose and lysine at 90 °C when the metal was added.

b The total reaction time was 6 hours.

c The content of the final product determined by ICP-OES.

d The UV absorbance at 420 nm of the final reaction solution.


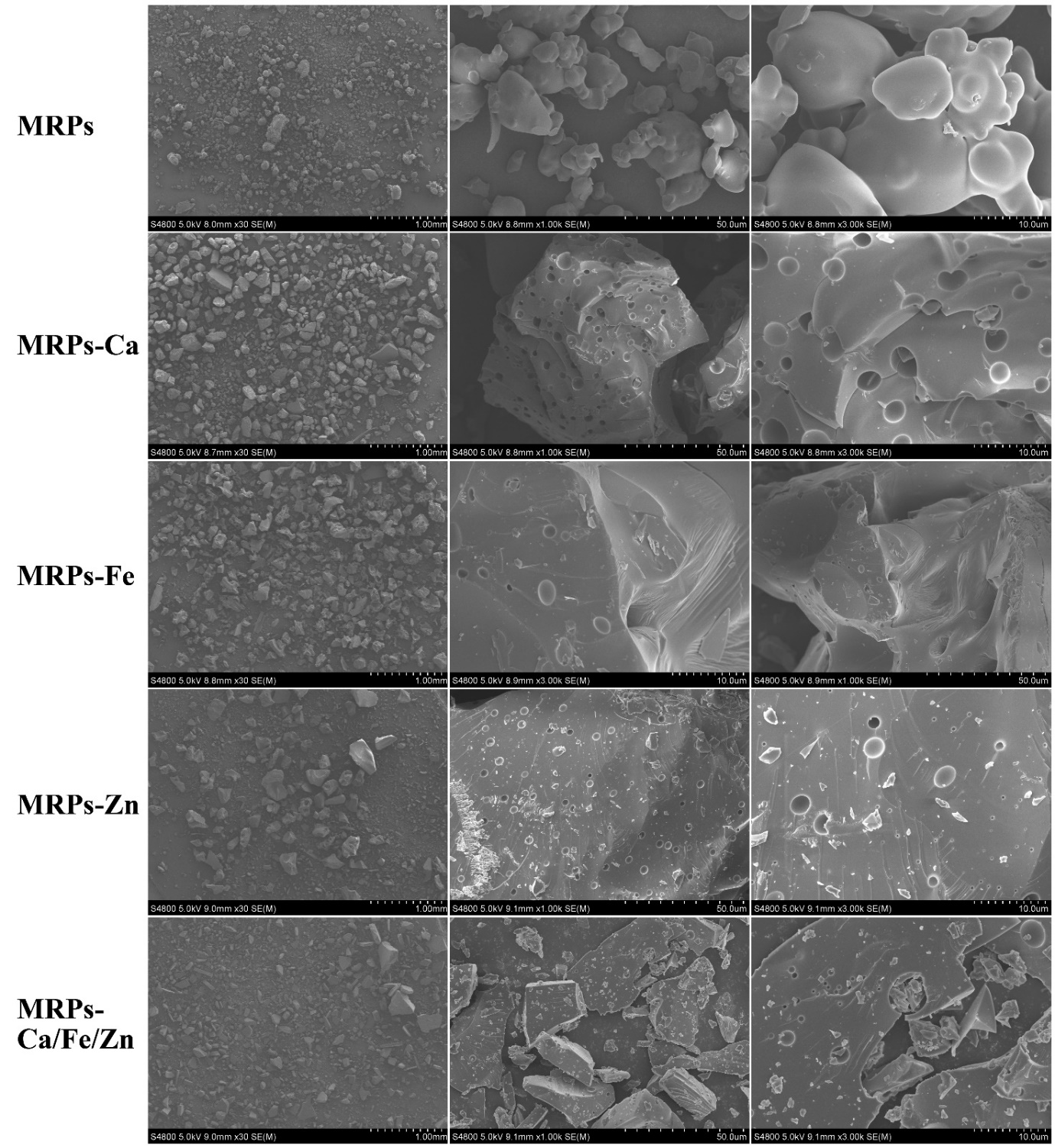


**Figure S2.** SEM images of the complexes.

**Table S2.** Zeta-potential of the complexes.

|  | MRPs | MRPs-Ca | MRPs-Fe | MRPs-Zn | MRPs-Ca/Fe/Zn |
| --- | --- | --- | --- | --- | --- |
| ζ-potential (mV) | 0.02 | -4.06 | 1.38 | 3.11 | 1.30 |


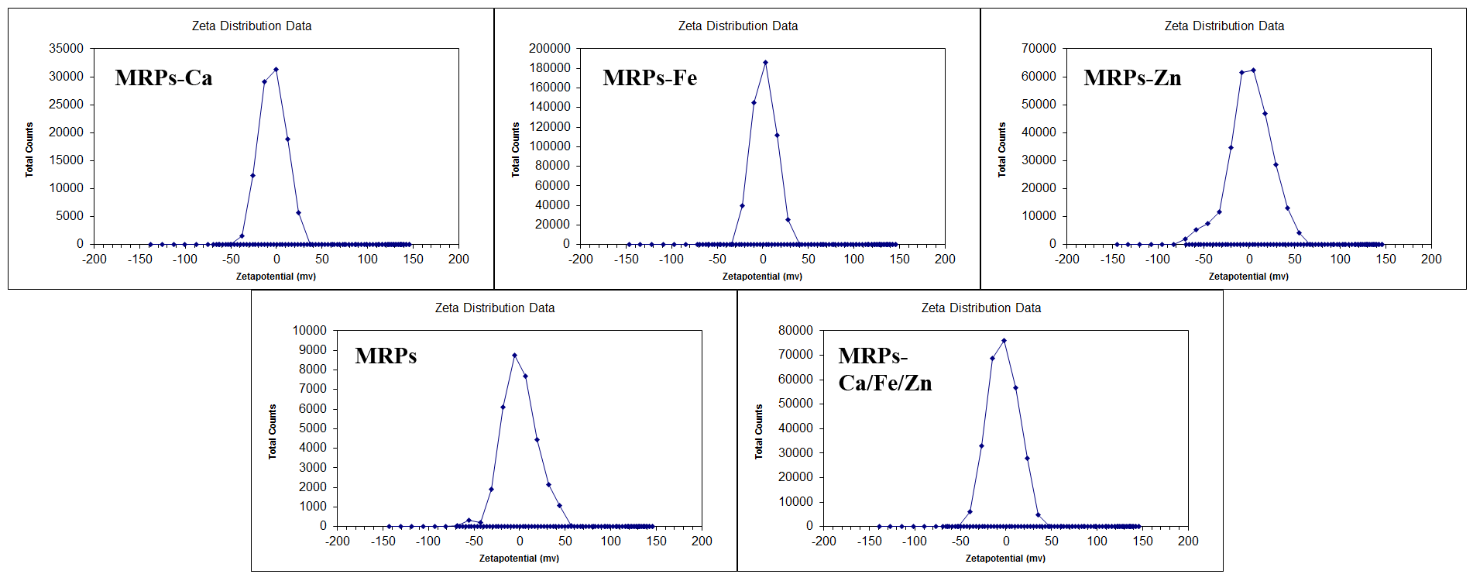


**Figure S3.** Zeta-potential distribution of the complexes.


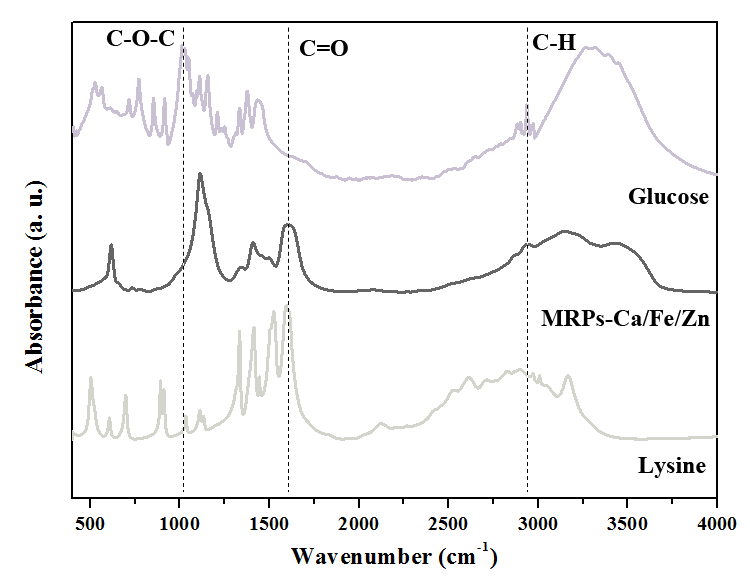


**Figure S4.** FTIR spectra of glucose, lysine, and MRPs-Ca/Fe/Zn.


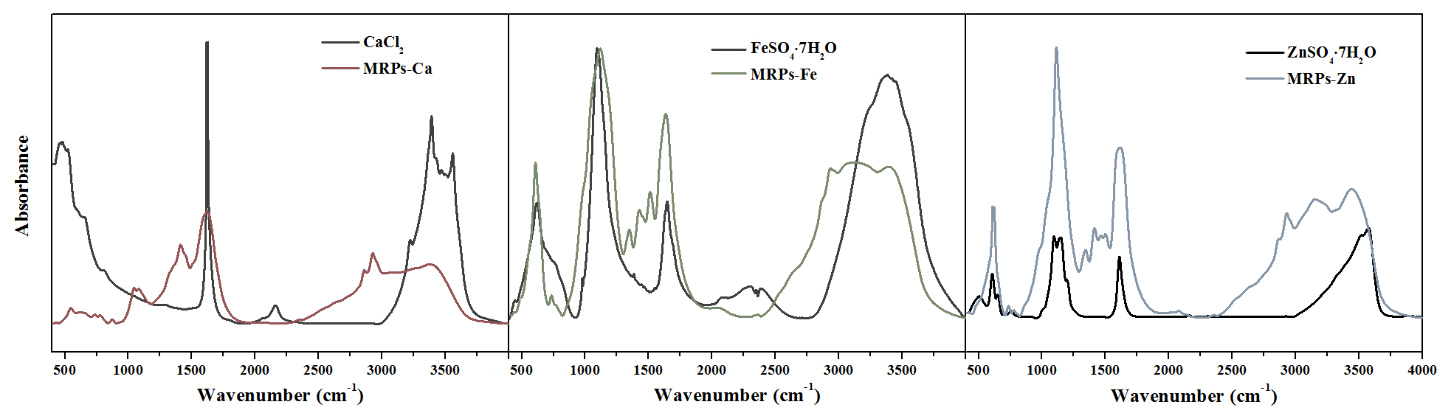


**Figure S5.** FTIR spectra of MRPs-Ca, MRPs-Fe, MRPs-Zn, and their inorganic raw materials.


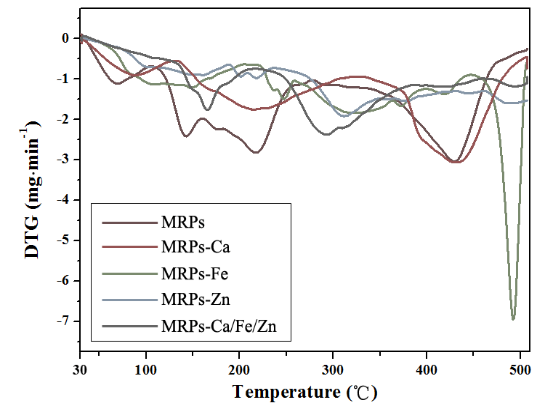


**Figure S6.** The DTG analysis of the MRPs and the complexes.

**Table S3.** Antioxidative activity of the complexes determined by the UV assay.

| Antioxidant activity | Samples |  |  |  |  |  |
| --- | --- | --- | --- | --- | --- | --- |
|  | MRPs | MRPs-Ca | MRPs-Fe | MRPs-Zn | MRPs-Ca/Fe/Zn | Standard^d^ |
| FRAP^a^ | 0.86±0.077 | 0.695±0.113 | 1.644±0.161 | 0.203±0.034 | 0.928±0.152 | 1.291±0.19 |
| DPPH^b^ | 0.104±0.027 | 0.116±0.053 | 0.167±0.021 | 0.486±0.044 | 0.176±0.042 | 0.086±0.028 |
| ABTS^c^ | 0.058±0.039 | 0.061±0.021 | 0.127±0.045 | 1.431±0.037 | 0.208±0.03 | 0.086±0.022 |

a Data expressed as absorbance at λ of 593 nm.

b Data expressed as absorbance at λ of 515 nm.

c Data expressed as absorbance at λ of 734 nm.

d Vitamin C at concentration of 1 mg·ml^-1^.


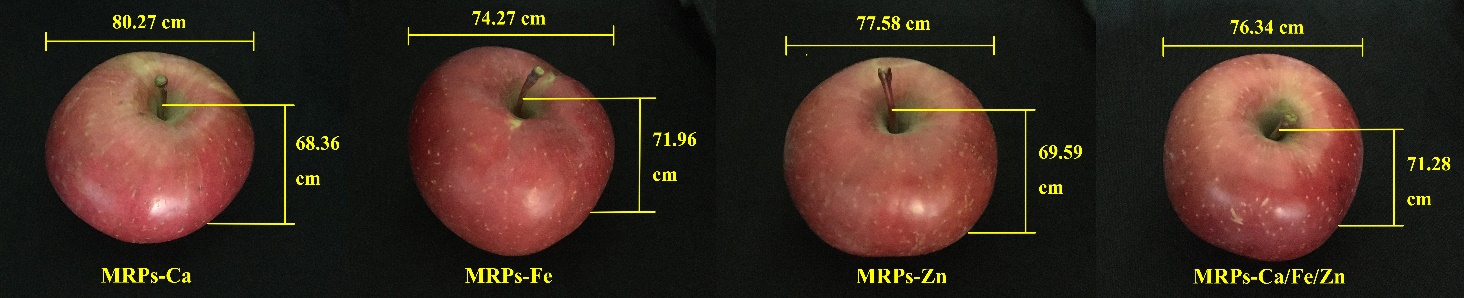


**Figure S7.** Harvested apple fruits treated by MRPs-Ca, MRPs-Fe, MRPs-Zn, and MRPs-Ca/Fe/Zn with high concentration (1%).

**Table S4.** SSC, TA, weight and firmness of the samples.

| Treatment | SSC (%) | TA (%) | Weight (g) | Firmness (kg·cm^-2^) |
| --- | --- | --- | --- | --- |
| T1 | 16.55±0.18 | 0.23±0.02 | 215.05±14.32 | 8.84±1.03 |
| T2 | 16.4±0.31 | 0.22±0.04 | 243.26±5.33 | 8.91 ±0.58 |
| T3 | 16.1±0.28 | 0.21±0.01 | 236.5±4.02 | 8.87±0.45 |
| T4 | 17.4±0.27 | 0.24±0.03 | 199.48±17.44 | 8.09 ±0.76 |
| T5 | 16.33±0.17 | 0.23±0.01 | 210.72 ±24.32 | 8.67 ±0.91 |
| T6 | 16.3±0.29 | 0.2±0.02 | 205.48±18.64 | 9.12±0.73 |
| T7 | 16.5±0.23 | 0.23±0.04 | 169.26±14.5 | 9.33 ±1.32 |
| T8 | 17.3±0.51 | 0.20±0.04 | 147.25±10.83 | 9.28 ±0.57 |
| T9 | 17.1±0.13 | 0.18±0.02 | 183.33±24.12 | 9.89±0.81 |
| Control | 15.8±0.13 | 0.26±0.01 | 192.85±15.89 | 10.05±0.67 |

**­­­**

| Treatment | Flesh (g·kg^-1^) | Peel (g·kg^-1^) | Leaf (g·kg^-1^) |
| --- | --- | --- | --- |
| T1 | 1.07±0.19 | 1.77±0.15 | 23.01±2.2 |
| T2 | 1.13±0.2 | 2.28±0.28 | 23.12±0.8 |
| T3 | 1.37±0.05 | 2.4±0.09 | 24.96±0.5 |
| T4 | 1.11±0.02 | 2.4±0.08 | 23.69±0.52 |
| T5 | 1.46±0.09 | 2.41±0.2 | 24.82±0.85 |
| T6 | 1.93±0.11 | 2.56±0.3 | 26.27±0.38 |
| T7 | 1.37±0.17 | 2.33±0.1 | 24.72±0.69 |
| T8 | 1.92±0.3 | 2.82±0.12 | 26.42±0.88 |
| T9 | 2.57±0.28 | 2.82±0.2 | 26.61±1.2 |
| Control | 1.29±0.09 | 2.51±0.12 | 24.09±1.12 |

**Table S5.** Content of N of the samples.

**Table S6.** Content of mineral nutrients of the samples.

| Treatment | Content | | | | | | | | |
| --- | --- | --- | --- | --- | --- | --- | --- | --- | --- |
|  | Ca (g·kg^-1^) | | | Fe (g·kg^-1^) | | | Zn (g·kg^-1^) | | |
|  | Flesh | Peel | Leaf | Flesh | Peel | Leaf | Flesh | Peel | Leaf |
| T1 | 0.34  ±0.14 | 0.43  ±0.1 | 11.33  ±1.53 | 5.49  ±2.46 | 42.68  ±4.46 | 0.18  ±0.03 | 2.42  ±0.16 | 2.79  ±1.46 | 138.03  ±38.14 |
| T2 | 0.46  ±0.15 | 0.56  ±0.07 | 12.7  ±0.87 | 20.4  ±4.17 | 48.95  ±5.17 | 0.18  ±0.04 | 2.74  ±0.21 | 4.65  ±2.22 | 139.54  ±30.25 |
| T3 | 0.74  ±0.18 | 0.66  ±0.09 | 13.03  ±1.93 | 24.95  ±2.83 | 68.8  ±6.33 | 0.27  ±0.12 | 3.06  ±0.34 | 6.97  ±2.38 | 251.43  ±40.93 |
| T4 | 0.35  ±0.15 | 0.46  ±0.15 | 14.49  ±0.91 | 10.50  ±2.92 | 49.49  ±4.52 | 0.31  ±0.12 | 2.07  ±0.16 | 3.59  ±1.87 | 130.77  ±42.13 |
| T5 | 0.4  ±0.17 | 0.52  ±0.17 | 17.83  ±2.02 | 24.78  ±4.35 | 79.13  ±4.35 | 0.39  ±0.08 | 2.83  ±0.22 | 7.29  ±1.25 | 179.36  ±49.31 |
| T6 | 0.5  ±0.16 | 0.68  ±0.16 | 18.29  ±2.71 | 28.88  ±2.52 | 93.45  ±5.29 | 0.44  ±0.03 | 4.25  ±0.17 | 13.78  ±3.17 | 261.74  ±38.16 |
| T7 | 0.47  ±0.21 | 0.41  ±0.21 | 18.35  ±1.53 | 24.95  ±2.5 | 85.64  ±8.45 | 0.35  ±0.06 | 2.62  ±0.12 | 3.75  ±2.44 | 126.54  ±18.4 |
| T8 | 0.54  ±0.18 | 0.64  ±0.08 | 19.01  ±1.14 | 35.49  ±2.46 | 99.97  ±4.11 | 0.54  ±0.06 | 2.79  ±0.18 | 10.23  ±2.49 | 357.22  ±32.38 |
| T9 | 1.05  ±0.2 | 0.57  ±0.2 | 20.54  ±2.08 | 42.06  ±4.95 | 137.72  ±7.85 | 0.85  ±0.1 | 3.56  ±0.34 | 19.78  ±2.26 | 377.59  ±59.82 |
| Control | 0.5  ±0.18 | 0.58  ±0.1 | 10.7  ±1.15 | 12.84  ±2.91 | 44.56  ±3.51 | 0.14  ±0.02 | 2.46  ±0.14 | 3.06  ±2.03 | 32.24  ±9.42 |
